# Digoxin-amiodarone Combination is Associated With Excess All-cause Mortality in Patients With Atrial Fibrillation

**DOI:** 10.1038/s41598-020-61065-4

**Published:** 2020-03-05

**Authors:** Jiun-Yang Chiang, Pau-Chung Chen, Yao-Hsu Yang, Chin-Hao Chang, Fang-Ying Chu, Jien-Jiun Chen, Cho-Kai Wu, Juey-Jen Hwang, Fu-Tien Chiang, Lian-Yu Lin, Jiunn-Lee Lin

**Affiliations:** 1 Division of Cardiology, Department of Internal Medicine, National Taiwan University Hospital Taipei, Taiwan and National Taiwan University College of Medicine, Graduate Institute of Clinical Medicine, Taipei, Taiwan; 2 Department of Occupational Medicine and Industrial Hygiene, National Taiwan University College of Medicine and Hospital, Taipei, Taiwan; 3 Department of Traditional Chinese Medicine, Chang Gung Memorial Hospital, Chia-Yi, Taiwan; 4 Department of Medical Research, National Taiwan University Hospital, Taipei, Taiwan; 5 Institute of Epidemiology and Preventive Medicine, College of Public Health, National Taiwan University, Taipei, Taiwan; 6 Division of Cardiology, Department of Internal Medicine, National Taiwan University Hospital Yun-Lin Branch, Yun-Lin, Taiwan; 7 Division of Cardiology, Department of Internal Medicine, National Taiwan University College of Medicine and Hospital, Taipei, Taiwan; 8 Division of Cardiology, Department of Internal Medicine, Fu-Jen Catholic University Hospital, New Taipei, Taiwan; 9 Division of Cardiology, Department of Internal Medicine, Taipei Medical University - Shuang Ho Hospital, Ministry of Health and Welfare, New Taipei City, Taiwan

**Keywords:** Cardiology, Interventional cardiology

## Abstract

Combination use of digoxin and other medications might lead to worse outcomes in patients with atrial fibrillation (AF). We sought to investigate whether digoxin-amiodarone combination would lead to worse outcome than digoxin alone in patients with AF. Adult patients with AF and received digoxin treatment from random samples of 1,000,000 individuals covered by the National Health Insurance in Taiwan were included. Baseline characteristics including risk factors and medications were matched by propensity score (PS) in those with and without addition of amiodarone treatment. A total of 5,040 AF patients taking digoxin therapy was included. PS matching identified 1,473 patients receiving digoxin-amiodarone combination and 2,660 patients receiving digoxin with a median follow-up of 1,331 days. Digoxin-amiodarone combination was associated with increased all-cause mortality (adjusted hazard ratio (HR): 1.640, 95% confidence interval (CI): 1.470–1.829, P < 0.001). The risk of mortality increased regardless of duration of combination. Risk of sudden cardiac death was not increased in the combination group (HR: 1.304, 95% CI: 1.049–1.622, P = 0.017). Death due to non-arrhythmic cardiac disease, cerebrovascular disease, and other vascular disease were higher in the combination group than the digoxin group. In conclusion, in patients with AF, digoxin-amiodarone combination therapy is associated with excess mortality than digoxin alone.

## Introduction

Digoxin is one of the oldest drugs in cardiovascular (CV) medicine, traditionally used in treating patients with atrial fibrillation (AF) and heart failure (HF)^1^, and one of the most frequently prescribed drugs in AF. In the Stroke Prevention using an ORal Thrombin Inhibitor in atrial Fibrillation (SPOTIF) study, 53% of patients were taking digoxin^2^. Digoxin is effective for long-term rate control at rest through slowing down atrioventricular conduction^3^. However, from meta-analysis and cohort study, use of digoxin might be associated with excess mortality in AF patients^2,4,5^.

In clinical practice, digoxin is frequently used in combination with other drugs, and many drugs interact with digoxin^6^. This may cause serum digoxin concentration (SDC) to exceed its therapeutic range, and according to the Digitalis Investigation Group (DIG) trial^7^, higher SDC resulted in less neurohormonal-inhibiting properties and higher rate of CV and all-cause mortality. Therefore, when interpretation of harmful effect of digoxin, concomitant drugs in use and their interactions with digoxin should be taken into consideration.

Dronedarone and amiodarone are two frequently concomitantly used drugs for rhythm control in patients with AF^8^. In the Permanent Atrial Fibrillation Outcome Study Using Dronedarone on Top of Standard Therapy (PALLAS) trial, elevated SDC by dronedarone was demonstrated^9^, and further investigation disclosed the potential harm of increased sudden death when dronedarone was used concomitantly with digoxin. Digoxin-dronedarone combination was discouraged afterward^8^. Whether patients with AF receiving digoxin-amiodarone combination therapy were in similar risk was unknown.

In this study, we carried out a nation-wide, population-based study to examine whether digoxin-amiodarone combination therapy was associated with increased mortality compared to digoxin alone^10^. Its impact on risk of sudden cardiac death (SCD) was also evaluated.

## Method

### Registry data sources

An universal national health insurance (NHI) program has been implemented in Taiwan since March 1995. Around 96% of the total Taiwanese population have been enrolled in the NHI program^11^ and by the end of 1996, the Bureau of NHI (BNHI) had contracted with 97% of hospitals and clinics throughout the nation^12^. BNHI accumulates all administrative and claim data for Taiwan.

The National Health Research Institute (NHRI) of Taiwan has cooperated with BNHI to establish NHI research databases. NHRI safeguarded the privacy and confidentiality of all beneficiaries. The health insurance data was transferred to health researchers by request after ethical approval had been obtained. To ensure the accuracy of the claim files, BNHI quarterly performed expert review on random samples of every 50–100 ambulatory and inpatient claims, and false report of diagnosis results in severe penalty from the BNHI^13^. Data for gender, birth date, medications, and diagnostic codes based on the International Classification of Diseases, Ninth Revision, Clinical Modification(ICD-9-CM; www.icd9data.com/2007) were retrieved for the analyses performed in this study. All research was performed in accordance with the relevant guidelines/regulations. The study protocol was approved by the research ethics committee of National Taiwan University Hospital. Because all the data was collected by National Health Research Institute, informed consent was waived by the research ethics committee of National Taiwan University Hospital for this study.

### Study population drug exposure and outcomes

For the current analysis, we used system sampling database from 1998 to 2009 with a total of 1,000,000 subjects. By using the ambulatory and inpatient claim data sets, we included subjects who were more than 18 year-old and who had been diagnosed AF after 1997. The index date was the date diagnosis of AF was made. Subjects who had taken digoxin during Jan. 1997 to Jan. 1998 or who had received digoxin and amiodarone subsequently were excluded. Subjects who had ever received digoxin were enrolled as digoxin group and subjects who had ever received digoxin and amiodarone simultaneously were enrolled as combination group. Cumulative duration of the use of digoxin for the digoxin group and digoxin-amiodarone combination for the combination group were collected.

### Comorbidities

Comorbidity was defined by diagnoses at hospital discharge or in clinic records. In our study population, we searched the database to see if they had hypertension, diabetes mellitus (DM) (ICD CM codes: 250.X, 249.X, A181), hyperlipidemia (ICD CM codes: 272.X, A189), coronary artery disease (CAD) (ICD CM codes: 411.X-414.X, V17.3, V81.0, A279), prior coronary intervention (ICD CM codes: 0066, 360.X), old myocardial infarction (MI) (ICD CM codes: 410.X, A270), transient ischemic accident (TIA) (ICD CM codes: 435.X, A299), ischemic stroke (ICD CM codes: 434.X, A293, A292), hemorrhagic stroke (ICD CM codes: 430.X-432.X), peripheral artery disease (PAD) (ICD CM codes: 250.7, 443.X, 444.2, A302, A301, A469), valvular heart disease (VHD) (ICD CM codes: 394.X-396.X, 398.X), chronic kidney disease (CKD) (ICD CM codes: 585.X-588.X), or cancer (ICD CM codes: 140.X-198.X, 200.X-208.X, 230.X-234.X). Charlson comorbidity index (CCI) of each patient were calculated. Conditions of CCI include dementia (ICD CM codes: 290.x, 331–331.2, 294), chronic pulmonary disease (ICD CM codes: 416.8–9, 490, 496), connective tissue disease (ICD CM codes: 710, 714, 725), peptic ulcer disease (ICD CM codes: 531–534), mild liver disease (without portal hypertension, includes chronic hepatitis) (ICD CM codes: 571.2, 571.4 × , 571.5, 571.6, 571.8, 571.9, 573), DM without end-organ damage (ICD CM codes: 250.0 × −250.3 × ), hemiplegia (ICD CM codes: 342, 344), moderate or severe renal disease (ICD CM codes: 581, 582, 583, 585, 586, 588, v42, v45.1, v56.x, 39.27, 39.42, 39.93–95, 54.98), tumor without metastases (ICD CM codes: 140.x-172.x, 174.x-195.x), leukemia (ICD CM codes: 204–208), lymphoma (ICD CM codes: 200, 202, 203), moderate or severe liver disease (ICD CM codes: 572.2–572.8, 456.0–456.2 × , 39.1, 42.91, 070), metastatic solid tumor (ICD CM codes: 196.x-199.x), and acquired immunodeficiency syndrome (ICD CM codes: 042.x-044.x). CHA_2_DS_2_-VASc (congestive heart failure, hypertension, age 65–74 years, diabetes mellitus, and vascular disease including myocardial infarction, coronary artery disease, and peripheral vascular disease [1 point for presence of each], and Stroke/TIA, age ≧ 75 years [2 points for presence of each]) of each patient was calculated.

### Outcomes

For study end point, we defined 2 outcomes. Outcome 1 was all-cause mortality and outcome 2 was SCD defined as resuscitation from ventricular tachycardia/ventricular fibrillation, cardiac arrest or cardioverter defibrillator implantation (with or without cardiac resynchronization therapy). In Taiwan, primary prevention of SCD in low left ventricular (LV) ejection fraction patients is not reimbursed by the national insurance, i.e, a patient must be a survivor of lethal ventricular arrhythmia before an cardioverter defibrillator implantation.

### Statistical analysis

All continuous variables are expressed as mean ± standard deviation and categorical variables as frequency (percentage). Propensity score (PS) matching was performed to account for differences in baseline characteristics between patients receiving combination therapy and those receiving digoxin only. PS was estimated for each patient using a logistic regression model in which the covariates were age, gender, history of Ischemic stroke/TIA, hemorrhagic stroke, CAD, coronary revascularization, old MI, PAD, HF, VHD, CKD, and cancer. Patients were matched on estimated PS using a nearest neighbor approach. Absolute standardized differences were calculated to evaluate the pre-match and post-match imbalance. Continuous variables were analyzed using t test, and categorical variables were compared using χ2 test where appropriate. A Cox proportional hazards model was used to calculate the hazard ratio (HR), using matched patients receiving digoxin only as the reference group. Two regression models were used. In model 1, age, gender, and risk factors, such as DM, HTN, and hyperlipidemia were included. In model 2, model 1 plus other comorbidities, including ischemic stroke/TIA, hemorrhagic stroke, CAD, coronary revascularization, old MI, PAD, HF, VHD, CKD, and cancer were adjusted. Subgroup analysis was performed based on age, sex, DM, CAD, and CHA_2_DS_2_VASc score^14^. Sensitivity analysis was conducted after excluding subjects with CAD, prior coronary intervention, old MI, or prior CABG. Analysis was performed with SPSS version 20 (International Business Machines Corp, NY). A 2-sided P value of < 0.05 was considered statistically significant.

## Results

**Figure 1:**
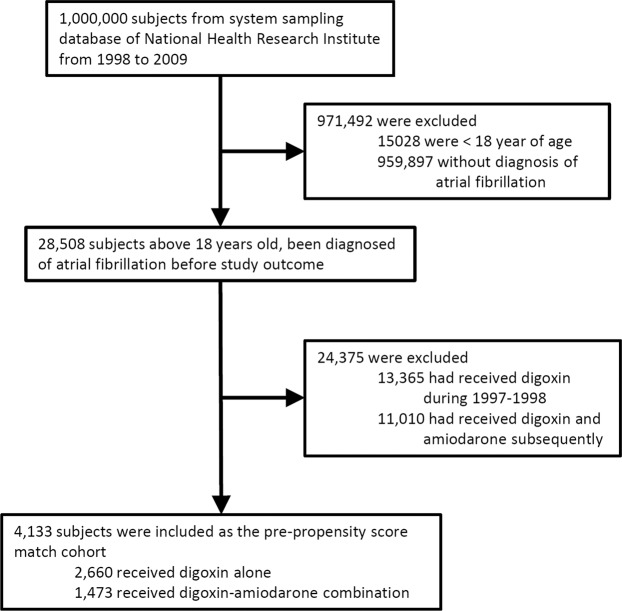
The algorithm for enrollment of the study subjects.

A total of 4,133 AF patients taking digoxin therapy was included as pre-matched population (Fig. 1). Among them, 2,660 patients received digoxin alone (the digoxin group), and 1,473 patients received digoxin-amiodarone combination (the combination group). Prevalence of DM (32.1% vs. 35.2%), hyperlipidemia (33.6% vs. 37.9%), CAD (55.3% vs. 58.9%), prior coronary revascularization (5.9% vs. 11.5%), old myocardial infarction (2.0% vs. 2.9%,), PAD (21.7% vs. 23.3%,), and VHD (7.9% vs. 9.8%,) were significantly higher in the combination group (Table 1). After PS match, mean age were 69 years old in both groups (Table 2). There is no difference in sex, prevalence of risk factors and comorbidities between the two groups. The absolute standardized differences of variables were all below 0.1 in the post-match population(Supplementary table 1 and supplementary figure 2).

**Table 1:** Basic demography of study subjects before propensity score match.

	Digoxin (N = 2660)	Combine (N = 1473)
**Risk factor**, %		
Age (years), mean ± SD	71.6 ± 12.7	70.9 ± 14.3
Gender, F	45.1	45.4
Hypertension	78.2	78.8
Diabetes mellitus	32.1	35.2*
Hyperlipidemia	33.6	37.9*
**Comorbidity**		
Ischemic stroke/TIA	31.4	32.8
Hemorrhagic stroke	4.3	4.9
Coronary artery disease	55.3	58.9*
Coronary revascularization	5.9	11.5*
Old myocardial infarction	2.0	2.9*
Peripheral artery disease	21.7	23.3*
Valvular heart disease	7.9	9.8*
Chronic kidney disease	11.1	14.2*
Cancer	13.2	15.8*
CHA_2_DS_2_VASC score, mean ± SD	4.0 ± 1.7	4.1 ± 1.7
CCI, mean ± SD	2.3 ± 2.1	2.7 ± 2.3*
**Medication**		
Aspirin	14.8	17.6*
Clopidogrel	3.3	6.8*
Warfarin	2.0	2.6
Betablocker	23.6	30.7*
ACEI	13.9	13.6
ARB	22.1	25.7*
DHP CCB	24.6	27.9*
Verapamil	2.0	4.1*
Diltiazem	9.0	17.2*
Spironolactone	5.9	7.6*
**Outcome**		
All-cause mortality	29.3	41.5*
Cardiac disease	4.1	5.2
Cerebrovascular disease	3.0	3.7
Other vascular disease	0.2	0.7*
Respiratory disease	5.8	10.7*
Infectious disease	10.0	14.3*
Cancer	3.2	2.9
Others	2.9	4.1
Sudden cardiac death, %	7.0	6.0
**Follow-up days, median (IQR)**	1424.0 (458.3–2981.8)	1135.0 (280.5–2497.5) *

TIA, transient ischemic attack; ACEI, angiotensin converting enzymes inhibitor; ARB, angiotensin receptor blocker; DHF CCB, dihydropyridine calcium channel blocker; AF, atrial fibrillation.

*P < 0.05 compared with digoxin group.

**Table 2:** Basic demography of study subjects after propensity score match.

Variable, %	Digoxin	Combine
**Risk factor**		
Age (years), mean ± SD	69.6 ± 13.4	69.7 ± 13.3
Female	43.9	42.9
Hypertension	76.7	77.4
Diabetes mellitus	31.8	31.4
Hyperlipidemia	39.3	39.6
**Comorbidity**		
Ischemic stroke/TIA	28.9	29.2
Hemorrhagic stroke	3.9	4.4
Coronary artery disease	51.6	53.9
Coronary revascularization	7.8	8.2
Old myocardial infarction	2.4	2.5
Peripheral artery disease	20.5	21.3
Valvular heart disease	5.6	5.9
Chronic kidney disease	12.3	13.3
Cancer	13.2	15.3
CHA_2_DS_2_VASC score	3.8 ± 1.8	3.8 ± 1.7
CCI, mean ± SD	2.3 ± 2.1	2.3 ± 2.1
**Medication**		
Aspirin	13.7	14.4
Clopidogrel	4.9	4.5
Warfarin	2.0	1.9
Betablocker	25.9	26.0
ACEI	9.7	10.0
ARB	24.4	24.8
DHP CCB	27.5	27.1
Verapamil	1.9	1.8
Diltiazem	8.8	8.6
Spironolactone	3.8	4.0
**Outcome**		
All-cause mortality	26.9	37.3^*^
Cardiac disease	3.7	5.0^*^
Cerebrovascular disease	2.9	3.0
Other vascular disease	0.1	0.8^*^
Respiratory disease	5.2	9.2^*^
Infectious disease	9.1	12.8^*^
Cancer	3.0	2.8
Others	2.9	3.8
Sudden cardiac death	6.7	5.9
**Follow-up days, median (IQR)**	1482.0 (496.0–3028.0)	1317.0 (426.0–2653.0)^*^

TIA, transient ischemic attack; ACEI, angiotensin converting enzymes inhibitor; ARB, angiotensin receptor blocker; DHF CCB, dihydropyridine calcium channel blocker; AF, atrial fibrillation.

*P < 0.05 compared with digoxin group.

All-cause mortality was significantly higher in the combination group (37.3% vs. 26.9%, P < 0.001). Death due to non-arrhythmic cardiac death (5.0% vs. 3.7%), other vascular disease (0.8% vs. 0.1%), respiratory disease (9.2% vs. 5.2%), and infectious disease (12.8% vs. 9.1%) occurred more often in the combination group than the digoxin group. The rate of SCD was similar between the two groups (6.7% and 5.9% for digoxin and combination group, respectively). There was a trend of increased SCD rate in combination group but did not reach statistical significance (8.2% vs. 10.0%, P = 0.068).

**Figure 2:**
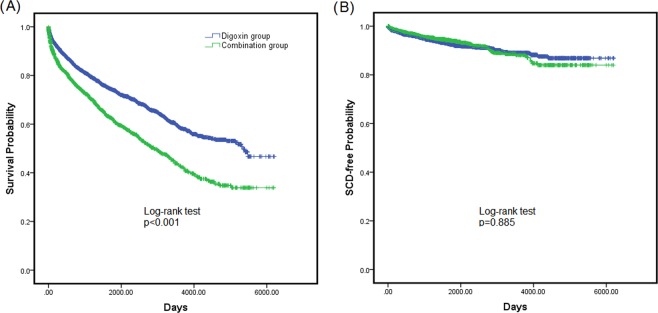
Kaplan–Meier curves shows the difference of survival rate between patients taking digoxin (blue) and digoxin-amiodarone combination (green) treatment (**1A**). The difference of sudden cardiac death rate between patients taking digoxin (blue) and digoxin-amiodarone combination (green) was also demonstrated (**1B**).

In the regression analysis (Table 3), two models were used to evaluate the HR of digoxin-amiodarone combination using digoxin treatment as reference. For all-cause mortality, the adjusted HR of digoxin-amiodarone combination was 1.768 with model 1 (95% confidence interval (CI) = 1.590–1.967, P < 0.001) and 1.640 with model 2 (95% CI = 1.303–1.736, P < 0.001). The HR remained significant in the sensitivity analysis (HR 1.770, 95% CI = 1.496–20.94, P < 0.001) For SCD, the adjusted HR were 1.031 (95% CI = 0.800–1.329, P = 0.813) with model 1, and 0.970 (95% CI = 0.748–1.258, P = 0.817) with model 2. The Kaplan-Meier curve revealed that the combination group had worse survival than the digoxin group, but there is no difference between the two groups regarding SCD-free survival (Fig. 2).

**Table 3:** Hazard ratios (95% C. I.) of all-cause mortality and sudden cardiac death for combination of digoxin and amiodarone vs. digoxin.

	HR	95% C.I.	P
**All-cause mortality**			
Model 1	1.768	1.590–1.967	<0.001
Model 2	1.640	1.470–1.829	<0.001
**Sudden cardiac death**			
Model 1	1.031	0.800–1.329	0.813
Model 2	0.970	0.748–1.258	0.817

Model 1, adjusted for age, gender and risk factors (DM, HTN, hyperlipidemia);

Model 2, adjust for age, gender, risk factors (hypertension, diabetes mellitus and hyperlipidemia), comorbidities (ischemic stroke/transient ischemic attack, hemorrhagic stroke, coronary artery disease, coronary revascularization, old myocardial infarction, peripheral artery disease, valvular heart disease and chronic kidney disease) and medications were adjusted in the model.

The combination group were divided into 3 subgroups based on duration of combination to see the effect of dose accumulation (Table 4). Regarding all-cause mortality, the HR significantly increased regardless of duration of combination, which were 1.771 (95% CI = 1.511–2.076, P < 0.001) for less than 14 days, 1.747 (95% CI = 1.510–2.023, P < 0.001) for between 14 to 60 days, and 1.735 (95% CI = 1.473–2.044, P < 0.001) for more than 60 days. For SCD, the HR was not increased regardless the duration subjects receiving the combination. The HR were 0.845 (95% CI = 0.551–1.295, P = 0.439) for less than 14 days, 1.266 (95% CI = 0.905–1.771, P = 0.168) for between 14 to 60 days, and 0.805 (95% CI = 0.516–1.257, P = 0.340) for more than 60 days.

**Table 4:** Hazard ratios (95% C. I.) of mortality and sudden cardiac death for different period of digoxin and combined digoxin and amiodarone usage after propensity adjustment.

	N	HR	95% C.I.	P
**All-cause mortality**				
≤14 days	522	1.771	1.511–2.076	<0.001
≤60 days	542	1.747	1.510–2.023	<0.001
>60 days	409	1.735	1.473–2.044	<0.001
**Sudden cardiac death**				
≤14 days	522	0.845	0.551–1.295	0.439
≤60 days	542	1.266	0.905–1.771	0.168
>60 days	409	0.805	0.516–1.257	0.340

Model adjusted for full model as model 2 in Table 1.

## Discussion

According to our study, digoxin-amiodarone combination is associated with excess all-cause mortality compared to digoxin alone. To the best of our knowledge, this is the first study to examine the real-world effect of digoxin-amiodarone rather than focusing on pharmacokinetics or few cases’ outcomes. The characteristics of the two study groups differed regarding risk factors of atherosclerosis and cardiac comorbidities. This reflected the real-world situation that patients with more comorbidities would receive amiodarone more often, and the more comorbidities a patient have, the higher chance drug-drug interaction occurs.

In our study, all-cause mortality increased regardless of duration of combination, including patients who received combination for less than 14 days. Prior case report showed that SDC started to fluctuate and elevate soon after amiodarone administration^15^, suggesting that the potential harm may begin early when patient started to receive digoxin-amiodarone combination.

### Interaction between digoxin and other drugs

Digoxin was recommended for treatment of HF and AF since its discovery two hundred years ago^16^. It slows AF rate by several proposed mechanisms^17^, and benefits patients with LV dysfunction, heart failure, or hemodynamic instability^8,18^. Patients with above condition often also received other medication therapy, and drug-drug interaction is an important issue for these patients since the therapeutic range of digoxin is narrow. Certain groups of patients require digoxin and amiodarone, for example, those with AF, acute coronary syndrome, or LV systolic dysfunction, digoxin and amiodarone are important choices of medication in this scenario. While using this combination, serum digoxin level would increase, and this may potentially lead to undesired outcome.

Plenty of medication were known to interact with digoxin. For example, bench and mice study had showed that adding quinidine would inhibited P-glycoprotein-mediated digoxin transport, and increased plasma digoxin concentration^19^. In cohort studies, addition of quinidine resulted in a mean 2.5-fold increase in SDC (from 0.98 ± 0.37 to 2.47 ± 0.71 ng/ml), P < 0.001) and the rate of digoxin toxicity^20^. Suggestion of a reduction of 30 to 50 percent of the digoxin dose when quinidine were used has been proposed^21^.

### The PALLAS

In the PALLAS trial, up to 33.6 (vs.32.5%) of patients in the trial were under digoxin therapy at baseline^9^. Because of potential of increased digoxin level caused by digoxin-dronedarone interaction, the investigators had been advised to use digoxin with caution and monitor serum levels closely. However, digoxin serum concentrations were still significantly higher in patients assigned to dronedarone therapy. This implied that despite judicious monitor and adjustment of digoxin use, adverse events of combination seemed not completely preventable. Among 1,070 patients receiving digoxin at baseline, there was a significant increase in CV deaths after add-on dronedarone (HR: 7.24, 95% CI: 1.65–31.67, P = 0.009). In contrast, among 2,166 patients not receiving digoxin at baseline, there was no significant increase in CV death with add-on dronedarone (HR, 0.76; 95% CI, 0.26–2.19; P = 0.61). Possible harmful effects of digoxin-dronedarone combination need be clarified in further study.

Few case reports also addressed the elevation of SDC and potentially fatal cardiac toxicity of digoxin when digoxin and dronedarone were used concomitantly, and the toxicity could occurred soon after starting digoxin^22^.

### Digoxin and amiodarone

Similar effects could be observed in digoxin-amiodarone combination. Animal and cohort study both showed that amiodarone could increase serum digoxin level, digoxin bioavailability, and showed a trend to prolong digoxin elimination half-life and decreased its renal clearance^23–26^. Case report in from pediatrics also demonstrated digoxin toxicity after initiation of amiodarone^27^. This had led to dose change or cessation of digoxin in substantial percentage of patients^28^. However, large scale long-term clinical outcome of digoxin-amiodarone combination was lacking.

In the analysis of cause of death, the combination group had more death other vascular disease before PS match. This is probably due to the fact that the combination group had more CAD, VHD, CKD, and more have received coronary revascularization. After PS match, the rate of death due to non-arrhythmic cardiac disease and other vascular disease were still higher in the combination group, suggesting that the combination may be associated with vascular events. Prior studies had pointed out increase thromboembolism associated with digoxin, possibly due to higher endothelium and platelet activation in patients receiving digoxin^29,30^. These findings are in accord with the results from post-hoc analysis of SPORTIF III and V and ROCKET AF^2,31^, which showed that digoxin is associated with increased myocardial infarction and vascular-related death^1^. Further investigation is needed to get more insight into the relationship between digoxin-amiodarone combination and endothelium and platelet activation.

### Limitations

There were several limitations in the study. First, selection bias remains despite PS match since it is impossible to include all the variables that may determine group membership from claimed database^32^. Higher rate of death due to respiratory disease and infectious disease might suggest that the combination group still represent a frailer group of patients despite PS match. Second, several important data were missing from claimed database, such as the SDC of each patient, renal function, left ventricle systolic function, drug compliance, and adequacy of anticoagulation. Prior investigators had added some of these determinants into regression model, and adjustment for these determinants, such as left ventricular function and serum creatinine level, did not change the finding that digoxin is associated with increased mortality. Third, the results may be affected by miss-coding. Because a prospective, randomized, placebo-controlled outcome studies examining the effect of digoxin-amiodarone combination therapy are not available, real world situation could only be reflected by registration study. If the combination is proved harmful, the number need to harm will be 15, and up to 0.4% of all patients could be effected^28^.

## Conclusions

In patients with AF, digoxin-amiodarone combination therapy is associated with excess mortality than digoxin alone. Death due to non-arrhythmic cardiac disease and vascular disease other than cerebrovascular disease was also higher among patients receiving this combination. These results consist with the observations of early case series, and further research is required to clarify reasons behind these findings.

## Perspectives

### Competency in medical knowledge

Digoxin-amiodarone combination should be discouraged in patients with AF.

### Competency in patient care

Patients with AF should be made aware that if they were receiving digoxin-amiodarone combination, a thorough discussion with their primary physician about the potential harmful effect should be made.

### Translational outlook

This is a retrospective cohort study. A randomized controlled study in the future would further verify the effect of digoxin-amiodarone combination.

## Supplementary information

Supplementary information
