# Supplementary material for: Digoxin-amiodarone Combination is Associated With Excess All-cause Mortality in Patients With Atrial Fibrillation
Source: Sci Rep. 2020 Mar 5;10:4101. doi: 10.1038/s41598-020-61065-4 (PMC7058044; doi:10.1038/s41598-020-61065-4)
Supplement: Supplementary file 1 — Supplementary information [file 41598_2020_61065_MOESM1_ESM.pdf]

Digoxin-amiodarone Combination is Associated With Excess All-cause Mortality in Patients With Atrial Fibrillation

Short title: Digoxin-amiodarone Combination in Atrial Fibrillation

Jiun-Yang Chiang, MD[1]; Pau-Chung Chen, MD, PhD[2]; Yao-Hsu Yang, MD, PhD[3]; Chin-Hao Chang[4]; Fang-Ying Chu[5]; Jien-Jiun Chen, MD[6]*; Cho-Kai Wu, MD, PhD[7]; Juey-Jen Hwang, MD, PhD[6]; Fu-Tien Chiang, MD, PhD[8]; Lian-Yu Lin, MD, PhD[7]*; Jiunn-Lee Lin, MD, PhD[9]

Supplementary figure 1. The absolute standardized difference of variables of pre- and post-propensity score match population

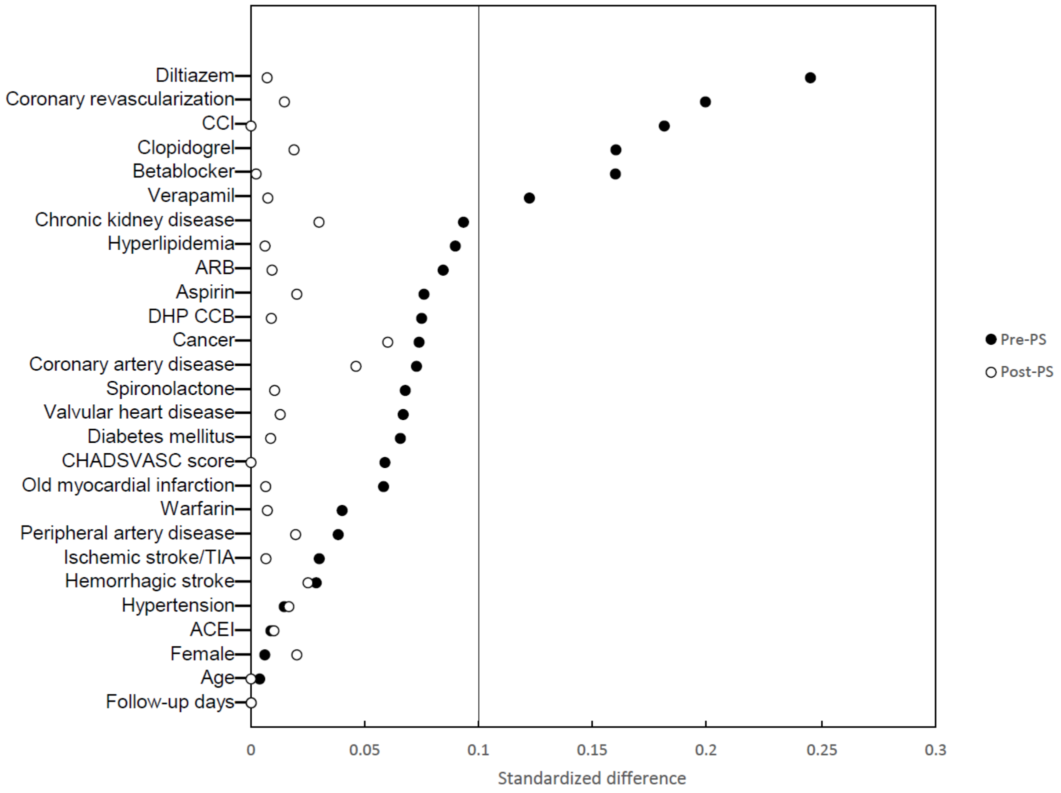

Supplementary Table 1. The absolute standardized difference of variables

	Absolute standardized difference	
Variable	Before PS match	After PS match
Diltiazem	0.24484922	0.007096394
Coronary revascularization	0.199685691	0.014744596
CCI	0.181630644	0
Clopidogrel	0.160348985	0.018900966
Betablocker	0.16015728	0.00228123
Verapamil	0.122350742	0.007421163
Chronic kidney disease	0.093359308	0.029935424
Hyperlipidemia	0.089811367	0.006138224
ARB	0.084488654	0.00928777
Aspirin	0.07604878	0.020144615
DHP CCB	0.075054003	0.008978749
Cancer	0.073892864	0.060102261
Coronary artery disease	0.072785221	0.046081961
Spironolactone	0.067798801	0.010330986
Valvular heart disease	0.066933989	0.012887114
Diabetes mellitus	0.065642103	0.008603842
CHADSVASC score	0.058823529	0
Old myocardial infarction	0.058241144	0.00646853
Warfarin	0.040033821	0.007232055
Peripheral artery disease	0.038322831	0.01967658
Ischemic stroke/TIA	0.029990897	0.006608066
Hemorrhagic stroke	0.028644595	0.025071714
Hypertension	0.014605242	0.016646978
ACEI	0.008711531	0.010067596
Female	0.006027287	0.020127652
Age	0.003797201	0
Follow-up days	0.000124914	7.13178E-05

Supplementary Table 2. The subgroup analysis of the study outcomes

All-cause mortality

	HR	CI	p
Age			
>65	1.642	1.461-1.846	<0.001
≤65	1.586	1.150-2.189	0.005
Sex			
Male	1.624	1.401-1.883	<0.001
Female	1.661	1.409-1.959	<0.001
CHA2DS2-VASc			
>4	1.665	1.424-1.946	<0.001
≤4	1.709	1.450-2.014	<0.001
CAD			
Yes	1.553	1.340-1.799	<0.001
No	1.756	1.486-2.074	<0.001
DM			
Yes	1.640	1.351-1.990	<0.001
No	1.965	1.431-2.697	<0.001

Sudden cardiac death

	HR	CI	p
Age			
>65	0.922	0.688-1.235	0.584
≤65	1.217	0.661-2.244	0.528
Sex			
Male	0.919	0.649-1.301	0.633
Female	1.067	0.713-1.596	0.752
CHA2DS2-VASc			
>4	0.946	0.633-1.414	0.787
≤4	1.000	0.690-1.450	0.999
CAD			
Yes	1.000	0.690-1.450	0.999
No	0.975	0.627-1.516	0.911
DM			
Yes	1.070	0.669-1.710	0.778
No	0.971	0.707-1.334	0.856
